# Manipulation of skyrmion motion by magnetic field gradients

**DOI:** 10.1038/s41467-018-04563-4

**Published:** 2018-05-29

**Authors:** S. L. Zhang, W. W. Wang, D. M. Burn, H. Peng, H. Berger, A. Bauer, C. Pfleiderer, G. van der Laan, T. Hesjedal

**Affiliations:** 1 Department of Physics, Clarendon Laboratory, University of Oxford, Parks Road, Oxford, OX1 3PU UK; 2 Faculty of Science, Ningbo University, 315211 Ningbo, China; 3 Magnetic Spectroscopy Group, Diamond Light Source, Didcot, OX11 0DE UK; 4 Crystal Growth Facility, Ecole Polytechnique Fédérale de Lausanne (EPFL), CH-1015 Lausanne, Switzerland; 5 Physik Department, Technische Universität München, James-Franck-Strasse 1, 85748 Garching, Germany

## Abstract

Magnetic skyrmions are particle-like, topologically protected magnetisation entities that are promising candidates as information carriers in racetrack memory. The transport of skyrmions in a shift-register-like fashion is crucial for their embodiment in practical devices. Here, we demonstrate that chiral skyrmions in Cu_2_OSeO_3_ can be effectively manipulated under the influence of a magnetic field gradient. In a radial field gradient, skyrmions were found to rotate collectively, following a given velocity–radius relationship. As a result of this relationship, and in competition with the elastic properties of the skyrmion lattice, the rotating ensemble disintegrates into a shell-like structure of discrete circular racetracks. Upon reversing the field direction, the rotation sense reverses. Field gradients therefore offer an effective handle for the fine control of skyrmion motion, which is inherently driven by magnon currents. In this scheme, no local electric currents are needed, thus presenting a different approach to shift-register-type operations based on spin transfer torque.

## Abstract (web summary)

Manipulation of skyrmions is one of the keys to achieving the skyrmion based spintronic devices. Here the authors show the skyrmions in Cu_2_OSeO_3_ can be rotated collectively with fixed velocity–radius relationship under a static magnetic field gradient which enables a local, Joule heat-free control of skyrmions.

## Introduction

Magnetic random access memory promises high-density, low-power-consumption devices for data storage applications. Initiated by the discovery of magnetic skyrmions, encoding magnetic bits by topologically protected magnetisation configurations presents an intriguing way to improve magnetic random access memory by increasing its robustness against superparamagnetism^1^. For example, in chiral magnets such as MnSi^2^, Fe_0.5_Co_0.5_Si^3^, FeGe^4^, Cu_2_OSeO_3_^5^ and Co_*x*_Zn_*y*_Mn_*z*_^6^, the individual skyrmions appear as spin swirls, which assemble into closed-packed, hexagonally ordered magnetic lattices. Such configurations are structurally incommensurate to the atomic lattice, energetically localised and stable^7,8^. Therefore, these magnetic structures are highly mobile, allowing for their electrical manipulation via spin transfer torque (STT) with ease^9–12^. Consequently, skyrmion-based racetrack memory^13^ was proposed^1,14–18^, being of great promise for future memory applications.

**Fig. 1:**
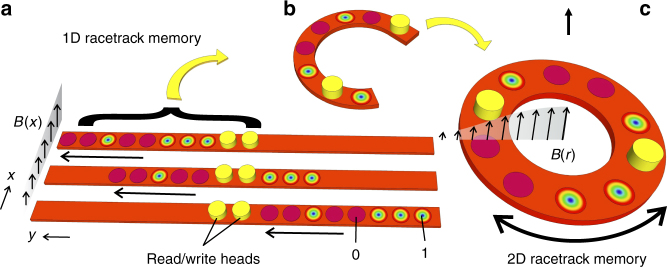
Circular skyrmion racetrack memory concept. **a** In the 1D skyrmion racetrack memory scheme, a skyrmion ensemble that encodes the information via the presence (1) or absence (0) of a skyrmion, is moved collectively in a shift register fashion. The skyrmions are written or read-out using local probes^20,21^, current-based injection elements^14,33^, or magnetoresistance elements based on the topological Hall effect^34^. For accessing every memory bit, the entire information stream has to be moved across the read/write head elements. **b** Morphing half of a 1D strip into a circular structure is the basis for the patterned 2D racetrack memory element shown in **c**. Here, the required rotation is controlled via an applied magnetic field gradient

The key operation of skyrmion racetrack memory is to move the entire magnetisation configuration collectively in a shift register fashion^13^, in order to read/write individual bits [(1) presence of a skyrmion; (0) absence or presence of a chiral bobber^19^] with a local probe^20,21^ as shown in Fig. 1a. Therefore, driving and controlling the skyrmion motion is the single most important task in the memory device. Up to now, current-driven skyrmion motion has been the most common method, as an ultralow current density of the order of 10^6^ A m^−2^ is sufficient to push the skyrmion lattice in chiral magnets^9,11^. However, such low current densities only allow for low-speed motion. To drive skyrmions at speeds comparable to ferromagnetic domain-wall motion (as used in standard racetrack memory), a similar current density has to be applied^1,11,12^, which is not beneficial in terms of energy consumption. As the electric current will eventually dissipate as Joule heat, it will be potentially detrimental for the stability of the local bits (i.e. the skyrmions and the ferromagnetic state) in terms of their positional order, especially since temperature stability and homogeneity are rather sensitive parameters in most skyrmion-carrying materials^2,10,22–24^. Moreover, the STT-based operation of skyrmion racetrack memory is only feasible in conductive materials. For semiconducting or insulating skyrmion materials, such a method is not applicable.

Here, we experimentally demonstrate that a non-contacting magnetic field gradient can be used to manipulate skyrmions efficiently, without the need for a driving electric current. In this way, the skyrmion material is not detrimentally perturbed by the source of manipulation, allowing for a local, Joule heat-free scenario. The required energy is largely reduced compared to the STT-scheme, and it is applicable to all skyrmion-hosting materials regardless of their conductivity. Indeed, transient field gradient-induced gyrotropic skyrmion bubble motion has been experimentally observed at GHz frequencies^25^. Here, we demonstrate an entirely different field gradient-induced effect by which the skyrmion lattice is collectively rotating at lower frequencies under a static magnetic field gradient.

## Results

### Skyrmion dynamics

The skyrmion dynamics is well-described in the Landau–Lifshitz–Gilbert micromagnetic framework^1^, and the centre of mass motion of a skyrmion was further generalised by Thiele^26^. Although skyrmions with different appearances are found in a variety of magnetic materials, the equations of motion share the same form in which the key quantity, the non-zero topological winding number *N*, is defined as $\frac{1}{{4\pi }}{\int} {\left( {{\textstyle{{\partial {\mathbf{m}}} \over {\partial y}}} \times {\textstyle{{\partial {\mathbf{m}}} \over {\partial x}}}} \right) \cdot {\mathbf{m}}{\mathrm{d}}x{\mathrm{d}}y}$ for a skyrmion in the $x$-$y$-plane, where **m** = (*m*_*x*_,*m*_*y*_,*m*_*z*_) is the local magnetisation unit vector. An integer $N$ gives rise to a finite gyromagnetic coupling vector ${\mathbf{G}} = (0,0, - 4\pi N/\gamma M_{\mathrm{S}}^2)$ ($M_{\mathrm{S}}$ is the saturation magnetisation), such that the equation of motion for a skyrmion without the presence of conduction electrons can be expressed as^10^:

1 $${\mathbf{G}} \times {\mathbf{v}}_d - {\cal D}\alpha {\mathbf{v}}_d = \nabla E$$

*E* is the total energy of the system, while the spatial gradient can be regarded as an effective force acting on the skyrmion, **v**_*d*_ is the drift velocity of the centre of mass of the skyrmion, *D* is the dissipative tensor, and *α* is the damping coefficient. The first term represents the Magnus force that is perpendicular to the skyrmion drifting velocity vector^9,10^, reminiscent to the behaviour of free electrons in a magnetic field, also referred to as the skyrmion Hall effect^27–29^. The second term denotes the dissipative drag force^1,9,10^. The drift velocity picks up a component that leads to a deviation from the main direction of motion (which is defined by **G**×**v**_*d*_; see Supplementary Methods 1). Here, a non-uniform perpendicular magnetic field [0, 0, *h*(*x*, *y*)] with an in-plane field gradient ∇*h* forces the skyrmions to move along contour lines (if damping is neglected)^30,31^. For example, a field gradient along the *x*-direction acts as an effective force exerted on the skyrmions along that direction. Consequently, the skyrmions pick up a velocity along the *y*-direction that is proportional to the amplitude of the gradient, as shown in Fig. 1a. In addition to this 1D racetrack memory scheme, a radial field gradient with concentric contour lines will lead to rotational motion of the skyrmions (see Supplementary Methods 1), allowing for a circular skyrmion racetrack memory scheme based on patterned rings (Fig. 1c). Note that for a practical device, one has to specifically take two effects into account. First, due to dissipation, the skyrmions will be on a spiralling path, and, second, the skyrmions will stop moving after a finite distance.

Conventional circular racetracks devices, based on domain-wall propagation, are already successfully used as multi-turn counters^32^. In both 1D^1,14–18^ and 2D racetrack devices, the skyrmion ensemble encoding the information is moved passed read/write heads^14,20,21,33,34^, however, in our case, relying on a magnetic field gradient.

### Resonant elastic X-ray scattering (REXS) experiment

**Fig. 2:**
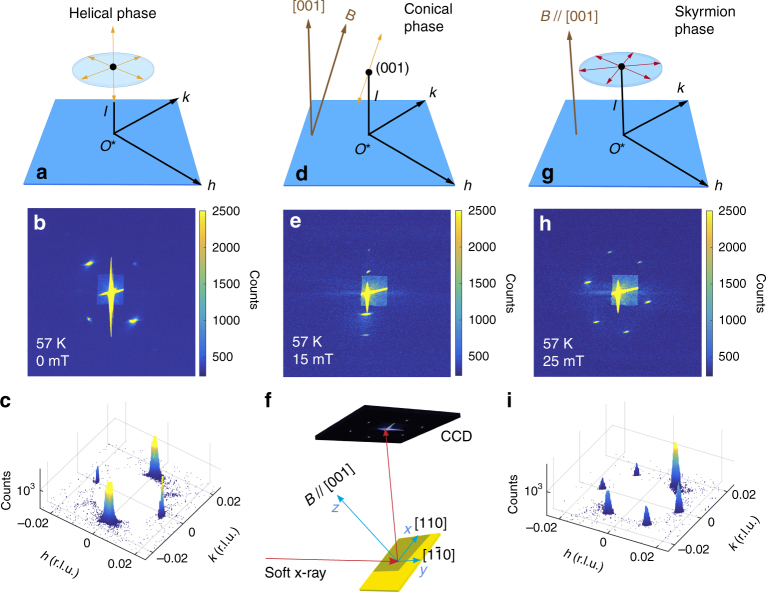
REXS results for the different magnetic phases of Cu_2_OSeO_3_ in the uniform magnetic field setup. The magnetically ordered wavevectors appear as satellites of the structural (0 0 1) peak in reciprocal space. **a**, **b** The helical phase is characterised by six wavevectors (shown in orange), of which four can be seen in the time-integrated CCD camera images (at 57 K, 0 mT). **c** The reciprocal space plot of the ($hk1$)-plane shows two sets of helical magnetic peaks. **d**, **e** For the conical state (at 57 K, 15 mT), the magnetic field has to be tilted to −17° in order to make the magnetic satellites visible. **g**–**i** The skyrmion lattice state (at 57 K, 25 mT) is characterised by six peaks. It is obtained by field-cooling from 65 to 57 K. The experimental setup and the scattering geometry are shown in **f**

In our experiment, the chiral magnet Cu_2_OSeO_3_ is used to demonstrate the skyrmion lattice rotation in a static radial field gradient. The material has two major advantages compared to other skyrmion systems. First, it is electrically an insulator, which means that the skyrmions can not be driven via STT^5^. Second, its relatively large crystal lattice constant allows for the observation of skyrmions using REXS^35,36^. REXS, with its CCD camera-limited minimum exposure time of ~1 ms, is ideally suited for studying slow dynamics in situ. Figure 2f shows the experimental geometry, in which a (001)-oriented single crystal is placed on the goniometer as sketched. The distance between the two cylindrical magnets, shown in Fig. 2 and oriented *N* and *S* as shown, can be adjusted in order to vary the field strength. Permanent magnets produce in-plane magnetic field gradients owing to demagnetisation effects and fabrication-related deviations from the ideal single-domain behaviour. Therefore, depending on the cross-sectional area and geometry of the magnets, one can choose a magnet pair that minimises these deviations and produces a very uniform field (uniform setup), or one that produces well-defined circular contour lines within the sample (gradient setup). See Supplemental Fig. 1 for detailed field distribution maps of the gradient magnet and Supplementary Methods 2–4 for details about the sample, the REXS setup and REXS calculations, respectively.

Figure 2 shows REXS results characteristic for the different magnetic orders of the chiral magnet Cu_2_OSeO_3_. At 57 K and 0 mT, the multidomain helical phase is stabilised as the ground state, characterised by two sets of magnetic satellites around the structural (001) peak. They lock along *h*, *k* and *l* directions as shown in Fig. 2a–c. Using the uniform setup, and increasing the field to 15 mT, leads to a single-domain conical phase, in which the magnetic satellites align with the magnetic field. Figure 2d, e shows the conical diffraction pattern, obtained by titling the magnetic field by   −17°. Keeping ${\mathbf{B}}\parallel [001]$, while increasing the field to 32 mT, will lead to the single-domain skyrmion lattice state, as shown by the sixfold-symmetric diffraction pattern in Fig. 2g–i. The magnetic modulation wavevector has an amplitude of *q*_*h*_ ≈ 0.0158 r.l.u. (reciprocal lattice units) for the helical, conical, and skyrmion lattice states, in agreement with other experimental reports^36,37^. The skyrmion lattice state is stable in an uniform magnetic field, where one pair of the magnetic peaks is locking along $\left\langle {100} \right\rangle$. Once this phase is formed, the diffraction pattern only undergoes slight drifts within ±2° over time due to thermal fluctuation-induced motion.

### Magnetic field gradients

**Fig. 3:**
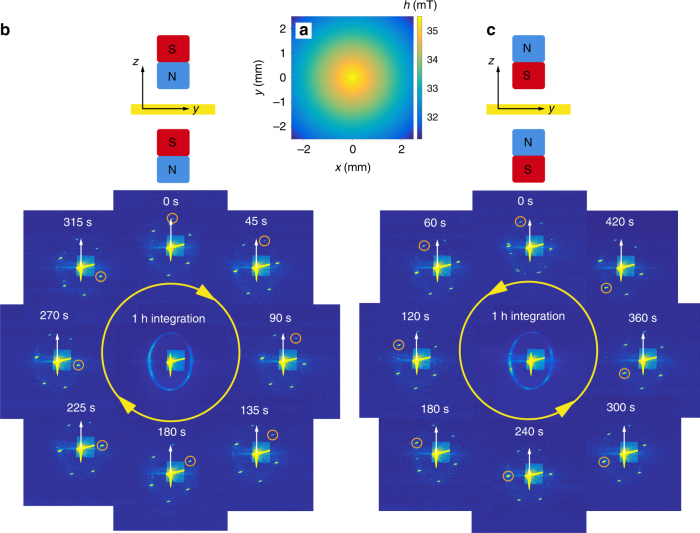
Skyrmion lattice rotation in magnetic field gradients. **a** The static external magnetic field ${\mathbf{B}} = [0,0,h(x,y)]$ is applied along the $z$-direction. The lateral distribution of *h*(*x*, *y*) is shown, in which the field is maximum in the centre and decreases outwards, leading to a radial field gradient in the plane. The sample is shown in yellow. **b** CCD camera snapshots taken at the indicated times during the field gradient-induced skyrmion lattice rotation. The rotation sense is clockwise. To better illustrate the motion, one of the magnetic satellites is marked by an orange circle, in comparison to the reference white arrow that is locked in the 12 o’clock direction. The integrated camera image over one hour is shown in the middle. **c** Anticlockwise skyrmion rotation obtained with the magnetic field applied in the opposite direction

We then use the gradient setup which produces a non-uniform perpendicular field with concentric contour lines, as shown in Fig. 3a. This static field distribution induces skyrmion lattice rotation. Figure 3b shows the rotation of the diffraction pattern through snapshots taken at different times as indicated. First, despite the variation in field strength, the field direction is always perpendicular to the sample surface over the entire sample area, i.e., ${\mathbf{B}}\parallel [001]$. The *h*(*x*, *y*) configuration has a circular shape, and radially decreases in field strength from the centre to the boundary. The field strength decrease of ~3 mT is less than the width of the skyrmion lattice phase pocket in the phase diagram of Cu_2_OSeO_3_. Second, the entire set of magnetic peaks rotates clockwise (for ${\mathbf{B}}\upharpoonleft \upharpoonright [001]$), in an almost steady motion. However, during their rotation, the magnetic satellites split into several subsets, each having a different angular velocity. In Fig. 3b, we track one of the magnetic peaks (marked by an orange circle). It rotates ~120° in 315 s, which is slow enough for REXS to track the trajectories of the individual diffraction spots quasi-continuously. The continuity of the rotation is also evidenced by the fact that the time-integrated pattern shown in the middle of Fig. 3a is ring-shaped. Third, when reversing the magnetic field direction while keeping the gradient the same, i.e. ${\mathbf{B}}\downharpoonleft \upharpoonright [001]$, the skyrmion lattice rotates counter-clockwise with a similar angular velocity, as is shown in Fig. 3c. It has to be stressed that the dependence of the rotation sense on the field direction excludes a thermal origin of the effect. In fact, we can exclude the presence of a sizeable temperature gradient, as reported in ref. ^24^ or other, X-ray-induced effects (see Supplementary Methods 5).

### Skyrmion lattice rotation dynamics

**Fig. 4:**
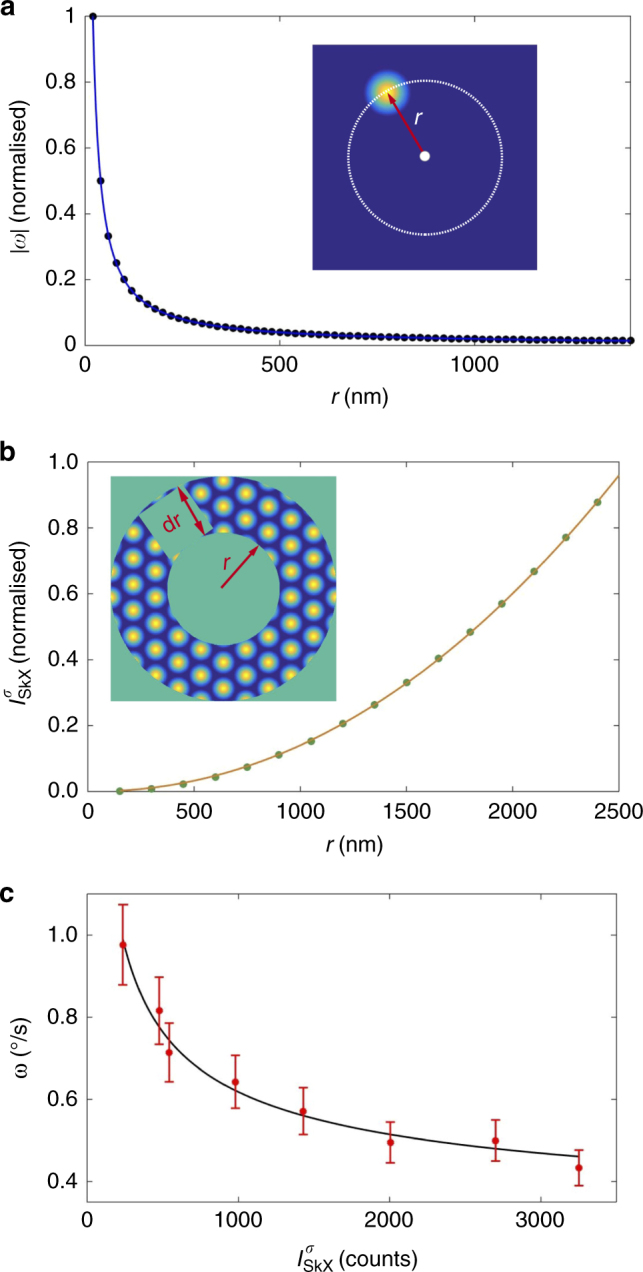
Universal velocity–radius relation for field gradient-induced skyrmion rotation. **a** Simulated angular velocity (*ω*) plot as a function of the distance of the rotating skyrmion from the centre (*r*) (shown as blue dots). The dependency follows a *ω* ~ *r*^−1^ relationship (blue line). **b** The calculated REXS intensity (using *σ*-polarised X-rays) shows a quadratic dependence on the track radius $r$ (track width ${\mathrm{d}}r$). The green dots are the numerical calculation results, which are best fitted with $I_{{\mathrm{SkX}}}^\sigma \sim r^2$ (orange line). **c** From the REXS data, the angular velocity *ω* of the rotating skyrmion track, and the resulting diffraction intensity $I_{{\mathrm{SkX}}}^\sigma$, can be acquired (red dots). The error bars represent the standard deviation from the mean value of the angular velocity, averaged over 1000 s. The measured $\omega (I_{{\mathrm{SkX}}}^\sigma )$ relation is in excellent agreement with the theoretical results, which predict $\omega \sim I_{{\mathrm{SkX}}}^\sigma$^−1/2^ (black line)

Interestingly, a pure sixfold-symmetric diffraction pattern, representative of a static, ordered skyrmion lattice, is never observed during the rotational motion. Instead, the diffraction peaks always split into multiple sets during the rotation. More importantly, the brighter the diffraction peak, the slower it rotates. This behaviour is indicative of a splitting of the skyrmion lattice into domains of different size that rotate at different speeds, consistent with our theoretical predictions shown in the Supplementary Methods 1. We find that for circular field contour lines, the skyrmion lattice rotates with varying angular velocity *ω*, depending on the distance *r* from the centre:

2 $$\omega = - \frac{\gamma }{{4\pi N(1 + \alpha ^2\eta ^2/N^2)}}\frac{1}{r}{\int} \frac{{\partial h}}{{\partial r}}(1 - m_z){\kern 1pt} {\mathrm{d}}x\;{\mathrm{d}}y$$

where *γ* is the gyromagnetic ratio, and *η* is the skyrmion shape factor that is close to unity. Equation (2) highlights several unique properties of field gradient-induced skyrmion rotation. First, the amplitude of the rotational angular velocity increases with increasing steepness of the gradient. Second, the rotation sense depends on the sign of the gradient, i.e. the skyrmions will rotate with opposite rotation sense if the direction of the magnetic field is reversed (consistent with the observations shown in Fig. 3). Third, for a linear field gradient, which is a good approximation for our experimental situation outside of the centre of the field distribution, Eq. (2) directly leads to $\omega \propto r^{ - 1}$. This approximation is sufficiently accurate for the presented analysis as confirmed by (micromagnetic) simulations presented in Fig. 4a. In other words, skyrmions rotate faster towards the centre of the magnetic field gradient, and slower further away. This divides the skyrmion lattice into different rings, similar to the tracks in a racecourse. Consequently, when the collective rotation starts, the long-range-ordered skyrmion lattice state breaks up into a multitude of circular domains, each with a different rotational speed. The resulting splitting of the diffraction peaks into multiple sets can be seen in the REXS data. The experimental REXS data shown in Supplementary Movies 1 and 2 are in excellent agreement with the REXS simulation data shown in Supplementary Movie 3.

In order to quantitatively measure the $\omega$-$r$ relationship, the scattering cross-section needs to be connected to the skyrmion lattice domain size. In our rotational scenario, the individually moving skyrmion lattice domains are rings, which are characterised by radius $r$ and ring width ${\mathrm{d}}r$, from which the total magnetic diffraction intensity $I_{{\mathrm{SkX}}}^\sigma$ can be calculated using $\sigma$-polarised incident X-rays^38^. As shown in Fig. 4b, $I_{{\mathrm{SkX}}}^\sigma$ is directly proportional to $r^2$ (see also Supplementary Methods 4), meaning that an outer domain track has a larger domain size, thus giving rise to brighter diffraction peaks. This straightforwardly leads to:

3 $$\omega \propto (I_{{\mathrm{SkX}}}^\sigma )^{ - 1/2}$$

In our REXS experiments, we are able to track many individual, sixfold-symmetric sets of peaks from different skyrmion lattice domains simultaneously, and to measure their rotating angular velocity and diffraction intensity. Figure 4c shows the measured $\omega (I_{{\mathrm{SkX}}}^\sigma )$ relationship (red dots), which is in excellent agreement with Eq. (3) (black line). This confirms that the observed field gradient-induced skyrmion motion follows the velocity–radius relation, similar to the semi-classical picture of atomic electron shells. This formation of concentric shells has also been observed, e.g. in dynamic processes of flux-line lattices in type II superconductors^39^ and superfluid helium^40,41^. When patterning a type II superconductor into a mesoscopic Corbino disk, the interaction between vortices, and between vortices and boundary, leads to a well-ordered vortex lattice, in many respects similar to the skyrmion lattice state. When applying a local current to the centre of the disk, the current density gradient drives the rotation of the vortex lattice, whereby the rotating lattice decomposes into rotating rings^42^.

### Dissipation effects

**Fig. 5:**
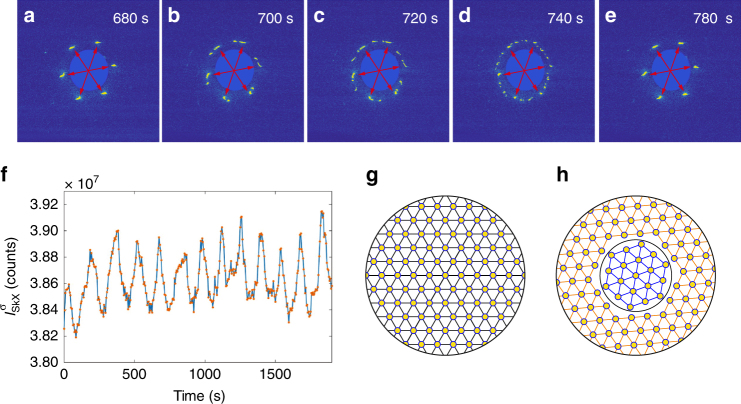
Field gradient-induced skyrmion lattice dynamics due to dissipation and temperature fluctuations. **a**–**e** Snapshots taken at different times during the rotation. The brightest skyrmion lattice peak set (marked by the red arrows) is locked after a certain period. The rest of the smaller domains keeps rotating for a finite lifetime, and the spots re-emerge shortly after having disappeared. **f** Total skyrmion lattice domain diffraction intensity as a function of time during the rotation. **g**, **h** Conceptual illustration of the rotation of skyrmion domains in circular racetracks. The annealed skyrmion lattice in **g** corresponds to the intensity maxima in **f**, and the minima to misaligned tracks illustrated in **h**

Next, we address dissipation effects in the context of skyrmion rotation. Due to damping, the rotation of the skyrmion lattice domains will only persist for a finite time. In particular, instead of constantly rotating along a circular loop, the skyrmions follow spiral trajectories (see Supplementary Methods 1). For a gradient field that is stronger in the centre and weaker further out, the skyrmions spiral outwards, and vice versa. When the rotation starts, the skyrmions rotate faster in the centre, and will gradually slow down while spiralling outwards. However, at the boundary of the sample, the skyrmion lattice is static^14^, which means that the outermost domains are not rotating. Moreover, skyrmions are repelling each other^14,15,43^, which prevents the inner domain tracks from spiralling out. In the model, this leads to a quasi-static rotation scenario which is reached after a certain period. As shown in Fig. 5a–e (see also Supplementary Movie 2), the brightest diffraction peaks (marked by red arrows), corresponding to the outermost shell of the domain tracks within the probing area (of 0.5 mm $\times$ 0.5 mm), are indeed becoming static after an initial rotation. This occurs immediately after the field gradient is applied. On the other hand, the inner shells of the skyrmion lattice domains keep rotating for a while, then suddenly disappear, joining the brighter domain peaks. This indicates that the smaller domains eventually spiral towards the outer shell, where they stop rotating. Nevertheless, we never observe a static diffraction pattern in an applied field gradient. In fact, we were able to observe the rotation for up to 6 days (duration of the beamtime) without reaching an equilibrium state. It is however important to note that any tracked domain peaks will disappear over time. Most importantly, at the same time, a new set of the skyrmion lattice domain peaks will emerge. As shown in Supplemental Movie 9, when confining the skyrmions to a patterned ring, the skyrmions will rotate under the field gradient without spiralling outwards or inwards. This may be the ideal design for a circular racetrack memory.

Figure 5f shows the total magnetic intensity as a function of time, obtained by integrating all skyrmion lattice domain contributions. The total magnetic scattering cross-section reflects the total number of skyrmions within the probed area^38^. As can be seen in Fig. 5f, the total scattering intensity is oscillating with time and varies by ~2.4%. Comparing the oscillation frequency with the series of diffraction patterns shown in Fig. 5a–e, it can be concluded that the measured intensity is large when the skyrmion lattices are all aligned, forming a single coherent lattice (giving six diffraction spots), and low when two or more sublattices are misaligned (giving rise to a multitude of sixfold spots). The loss in measured intensity points strongly towards a plastic rather than an elastic behaviour of the skyrmion lattice, in some ways similar to the plastic flow involving tearing in rotating superconducting vortex lattices^44^. At the boundaries of the rotating domain, where the relative velocity differences are large, the regular order of the lattices breaks down, leading to skyrmion or skyrmion-like structures which are no longer counted by integrating the diffraction intensity. Figure 5 shows a conceptual illustration of the domain rotation mechanism. As the skyrmions closer to the centre will rotate faster than domains further out, the rotating ensemble (shown in Fig. 5g) disintegrates into a shell-like structure of discrete circular racetracks as evidenced by the peak splitting [cf. Figure 5a–e], shown in Fig. 5h. The different angular velocities result in a domain boundary between the racetracks, leading to a reduced total scattering intensity as shown in Fig. 5f. Whenever the domain orientations coincide during the course of the rotation, the domain boundary disappears, leading to an annealing of the skyrmion lattice and a full recovery of the intensity.

## Discussion

Although the circular motion of the skyrmions is governed by the magnetic field gradient, the inherent source of energy of the skyrmion motion is, in the absence of other energy sources such as electric currents, the momentum carried by magnons due to temperature inhomogeneities across the sample surface^23,24^. As we can rule out direct soft X-ray-based heating effects (see Supplementary Methods 5 for details), we can estimate an upper bound for the temperature variation across the sample surface of <20 mK. Note that this gradient is orders of magnitude smaller than the gradients encountered in electron beam-based microscopy techniques, with which rotating skyrmion ratchet motion has been observed^24^. Nevertheless, owing to the remarkably small Gilbert damping parameter *α* of about $1 \times 10^{ - 4}$ at 5 K^45^, such small local temperature variations can be sufficient to drive the skyrmion motion for an extended period of time.

In summary, we have demonstrated that skyrmions can be manipulated with the help of a magnetic field gradient. Both velocity and direction of the moving skyrmions can be controlled in a defined way by changing the field parameters (see Supplementary Movie 7). First, in a practical skyrmion racetrack device, Oersted fields can be used to create a similar field gradient. Although Joule heating is inevitably generated in the coils, the required current densities are largely reduced compared with the those needed for direct, STT-driven skyrmion transport in metallic systems. This scheme also avoids local heating of the skyrmion-hosting material, and it can be applied to all skyrmion materials regardless of their conductivity. Second, the direction of the moving skyrmions and their velocity can be directly controlled via the field gradient parameters. It is worth noting that in our experiment, the observed angular velocity is rather slow (i.e. <1° s^−1^), as the observed skyrmion lattice domain tracks extend to a rather large radius on the mm scale. We also evaluated smaller domains as they appear in the centre of the field distribution (or in patterned nano-sized disks), and assuming a radius of the outermost track of *r* ≈ 0.5 mm, while *ω* ≈ 0.5° s^−1^, we obtain a linear velocity of *v* = 2*πωr* ≈ 1.6 × 10^6^ nm s^−1^. This linear velocity is universal for all domain tracks (due to the $\omega \propto r^{ - 1}$ relationship), and consequently also for skyrmions in linear racetracks (as shown in Fig. 1a and Supplemental Movie 10). Compared to typical velocities of 10^5^ nm s^−1^ for STT-induced skyrmion lattice motion in, e.g. FeGe above the critical current density of ~100 A cm^−2^ (ref. [11]), field gradient-induced motion is in fact a very promising route for practical devices (see Supplementary Methods 6). Moreover, in our experiment, the small applied gradient of less than 0.1 mT mm^−1^ was already sufficient to control the skyrmion motion. The magnitude of the gradient can be easily increased to 10’s of mT mm^−1^, and will thus proportionally enhance the rotational speeds to device-relevant values.

## Methods

Methods and any associated references are available in the Supplementary Information.

### Data availability

The data that support the findings of this study are shown in the Supplementary Information files and are further available from the corresponding author on request.

## Electronic supplementary material

Supplementary Information

Descriptions of Additional Supplementary Files

Supplementary Movie 1

Supplementary Movie 2

Supplementary Movie 3

Supplementary Movie 4

Supplementary Movie 5

Supplementary Movie 6

Supplementary Movie 7

Supplementary Movie 8

Supplementary Movie 9

Supplementary Movie 10
