# Supplementary material for: Manipulation of skyrmion motion by magnetic field gradients
Source: Nat Commun. 2018 May 29;9:2115. doi: 10.1038/s41467-018-04563-4 (PMC5974091; doi:10.1038/s41467-018-04563-4)
Supplement: Supplementary file 1 — Supplementary Information [file 41467_2018_4563_MOESM1_ESM.pdf]

# Manipulation of Skyrmion Motion by Magnetic Field Gradients

**Zhang et al.**

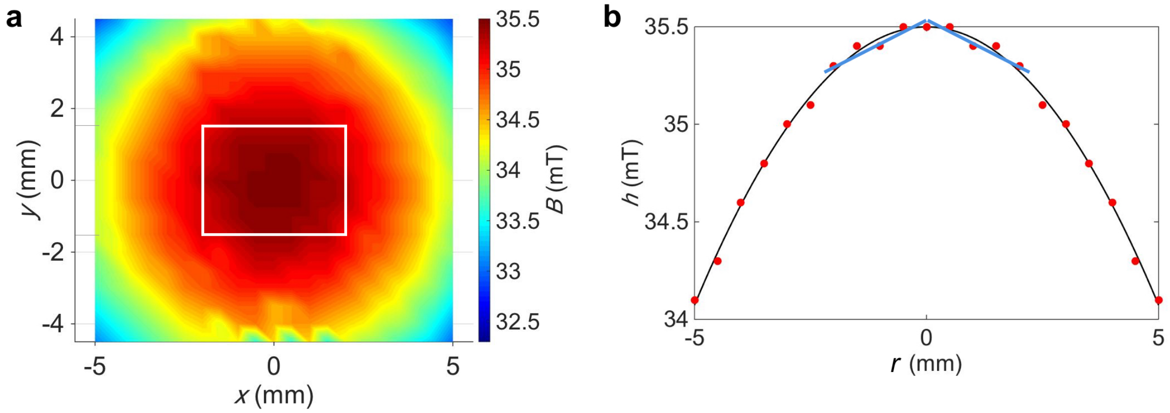


**Supplementary Figure 1 — Experimental determination of the field-gradient.** **a**, The in situ measured $z$-component of the non-uniform field distribution produced by the permanent magnet pair. The two-dimensional field map is performed by raster-scanning a Hall probe (probing area: 1.25 mm $\times$ 1.25 mm) using high-precision stepper motors (step size 0.05 mm). The (001)-oriented $Cu_2OSeO_3$ single crystal, measuring 4 mm $\times$ 3 mm, was placed in the centre of the field gradient. **b**, Line-scan using the Hall probe cutting across the centre of the sample position. The field is best fitted by a parabolic function, $h(r)$ [mT] $= 0.057\,r^2 + 35.50$ ($r$ in mm), as shown by the black line (red dots are measured points). The sample is located in the very centre of the field distribution where the slope is almost linear and $h(r)$ can be approximated by a linear function, $h(r)$ [mT] $= 0.10\,r + 35.52$ ($r$ in mm), as shown by the blue lines.

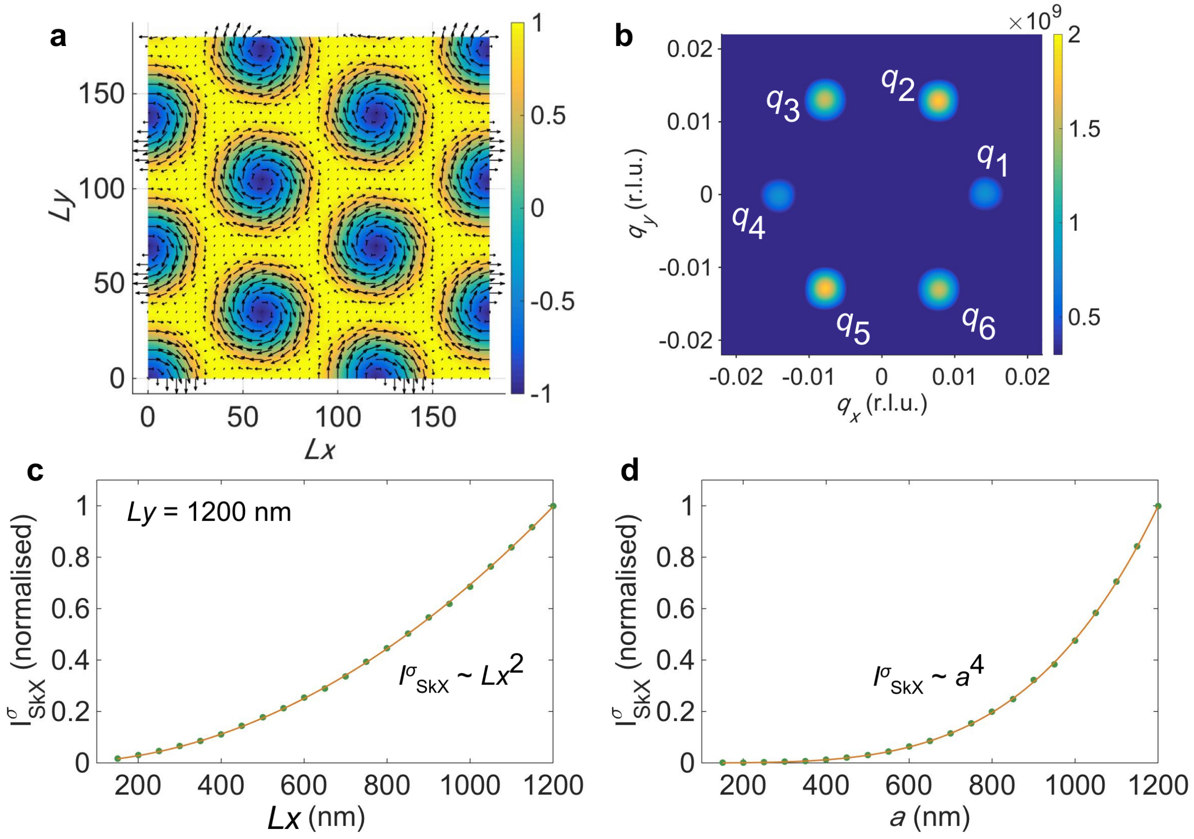


**Supplementary Figure 2 — Numerical calculation of the diffraction intensity as a function of geometrical parameters.** **a**, Square-shaped single domain skyrmion lattice. **b**, Simulated diffraction patterns using $\sigma$-polarised light. The six peaks are labelled $q_1, ..., q_6$. **c**, $I^{\sigma}_{\mathrm{SkX}}$ is the total peak intensity from the six peaks, corresponding to our measurement intensity. The plot shows the $I^{\sigma}_{\mathrm{SkX}} \sim S^2$ relationship. **d**, Representation of $I^{\sigma}_{\mathrm{SkX}}$ as a function of the side length $a$ of a square mesh.

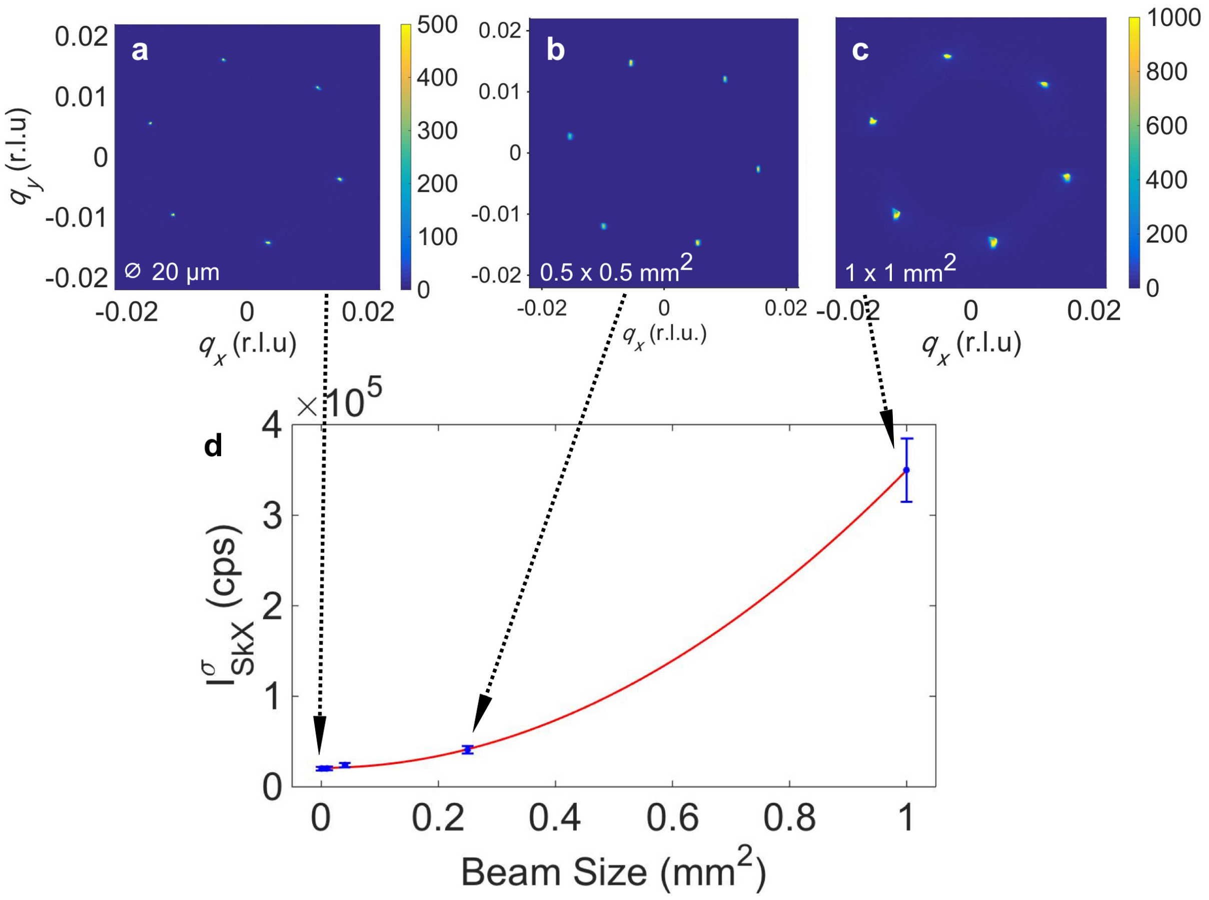


**Supplementary Figure 3 — Scattering intensity as a function of beam size. a-c**, Experimental scattering data using $\sigma$-polarised incoming x-rays with the beam area (i.e., beam size) given in the figure. **d**, Total scattering intensity obtained by integration over the six peaks, showing that $I^{\sigma}_{\mathrm{SkX}} \sim S^2$.

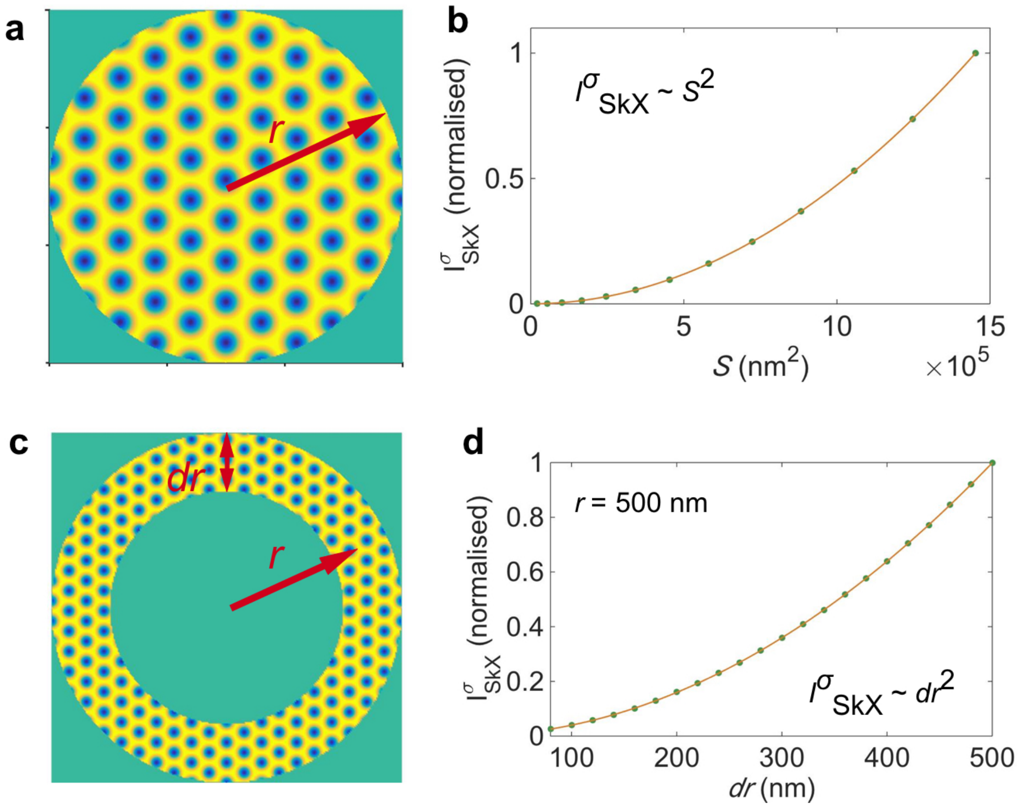


**Supplementary Figure 4 — Scaling of scattering intensity for a skyrmion disk and a ring.** **a**, Disk-shaped skyrmion lattice (radius $r$), and, **c**, circular segmented skyrmion lattice (radius $r$, width $dr$). **b**, The plots of $I^\sigma_{\mathrm{SkX}}$ again shows the $\propto S^2$ relationship for the disk. **d**, For the circular segment, we find $I^\sigma_{\mathrm{SkX}} \propto dr^2$ (for fixed $r$).

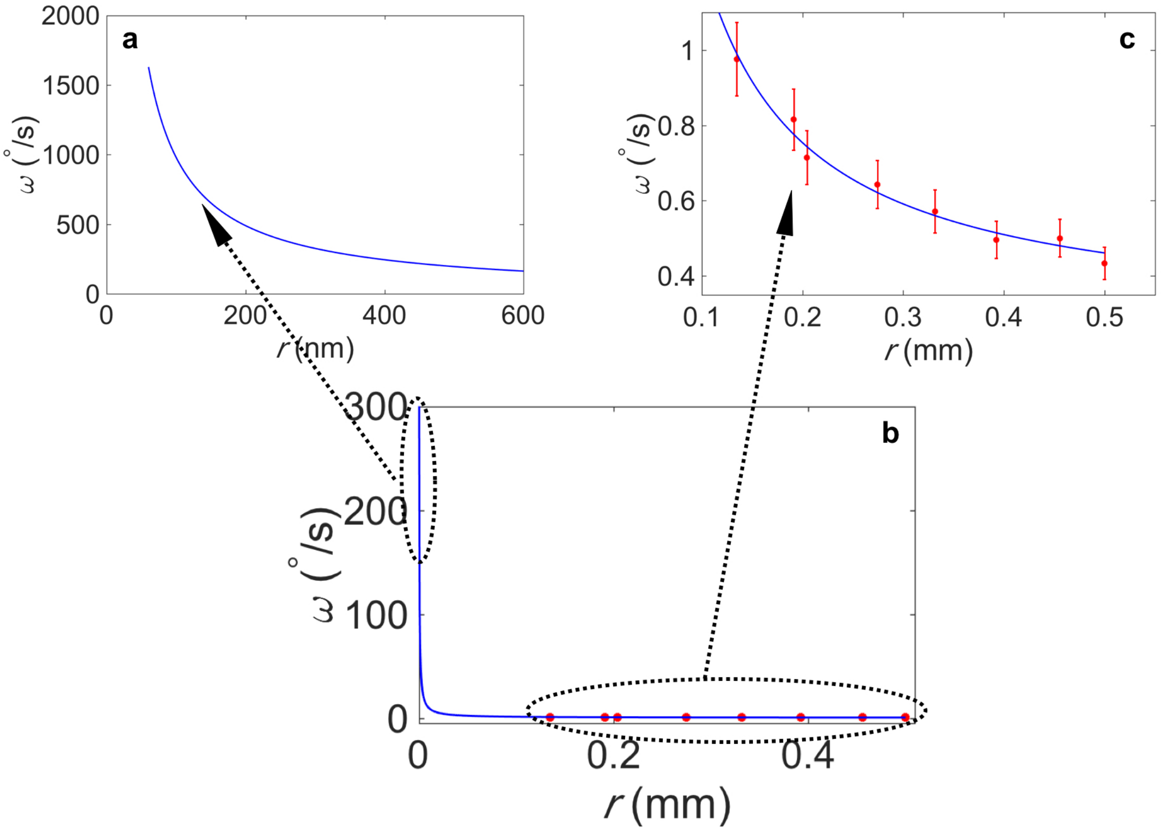


**Supplementary Figure 5 — Extraction of the $\omega(r)$ relationship from existing data and the well-established model.** **a**, Zoomed-in plot of the $\omega(r)$ relationship for small $r$. (Note that the $r$ scale is in nm). **b**, Overview plot, following $\omega \propto r^{-1}$. **c**, Zoomed-in plot for large $r$. The error bars represent the standard deviation from the mean value of the angular velocity, averaged over $1000\,\mathrm{s}$.

**Supplementary Methods 1. Skyrmion motion in a magnetic field gradient**

The dynamics of the magnetisation $\mathbf{m}$ is governed by the Landau-Lifshitz-Gilbert (LLG) equation

$$\frac{\partial \mathbf{m}}{\partial t} = -\gamma \mathbf{m} \times \mathbf{H}_{\text{eff}} + \alpha \mathbf{m} \times \frac{\partial \mathbf{m}}{\partial t}, \tag{1}$$

where $\gamma$ ($> 0$) is the gyromagnetic ratio and $\alpha$ is the Gilbert damping constant. The effective field $\mathbf{H}_{\text{eff}}$ associated with the micromagnetic energy $E$ is given by $\mathbf{H}_{\text{eff}} = -(\delta E/\delta \mathbf{m})/(\mu_0 M_s)$, where the energy $E$ in our studied system is given by

$$E = \int \left[ A(\nabla \mathbf{m})^2 - \mu_0 M_s \mathbf{m} \cdot (\mathbf{H}_0 + \mathbf{h}) + D\mathbf{m} \cdot (\nabla \times \mathbf{m}) \right] \mathrm{d}V \ , \tag{2}$$

where $\mathbf{H}_0$ is the constant external field and $\mathbf{h}$ represents the field gradient. Therefore, the corresponding effective field is $\mathbf{H}_{\text{eff}} = 2/(\mu_0 M_s)[A\nabla^2 \mathbf{m} - D\nabla \times \mathbf{m}] + (\mathbf{H}_0 + \mathbf{h})$. The static Zeeman field $\mathbf{H}_0$ stabilises the skyrmion phase. The skyrmion motion is affected by the presence of gradient field $\mathbf{h}$. To describe the dynamics of skyrmions in a field gradient, we use the approach developed by Thiele [1]. For the translational motion the rigid skyrmion model is used, i.e., we assume $\mathbf{m}(\mathbf{r}, t) = \mathbf{m}(\mathbf{r} - \mathbf{R}(t))$. Therefore, $\partial_t \mathbf{m} = -(\mathbf{v}_d \cdot \nabla)\mathbf{m}$ where $\mathbf{v}_d = \partial_t \mathbf{R}$. Following the treatment given in Ref. [2], we obtain

$$\mathbf{G} \times \mathbf{v}_d - \mathcal{D}\alpha \mathbf{v}_d = \nabla E = -\mathbf{F} \ , \tag{3}$$

where $\mathbf{G} = 4\pi Q \mathbf{e}_z$ with $Q = -(1/4\pi) \int \mathbf{m} \cdot (\partial_x \mathbf{m} \times \partial_y \mathbf{m}) \, dx \, dy$ the topological charge (skyrmion number). The tensor element $\mathcal{D}_{ij}$ is given by $\mathcal{D}_{ij} = \int (\partial_i \mathbf{m} \cdot \partial_j \mathbf{m}) \, dx \, dy$, and simplifies to $\mathcal{D}_{ij} = 4\pi \delta_{ij} \eta$ for the case that the shape factor of the skyrmion $\eta$ is close to unity. The force $\mathbf{F}$ induced by the field gradient is given by

$$F_i = -\gamma \int \mathbf{m} \cdot [\partial_i \mathbf{m} \times (\mathbf{m} \times \mathbf{h})] \, dx \, dy \ , \tag{4}$$

which can be simplified to $F_i = -\gamma \int (\mathbf{h} \cdot \partial_i \mathbf{m}) \, dx \, dy$.

For a single skyrmion moving in a field gradient of the form $\mathbf{h} = h(x)\mathbf{e}_z$, and by requiring that $m_z(\infty) = 1$, we arrive at

$$F_x = -\gamma \int (\partial_x h)(1 - m_z) \, dx \, dy \qquad \text{and} \qquad F_y = 0 \ , \tag{5}$$

where we have applied a partial integration. Therefore, the drift velocity $\mathbf{v}_d$ is given by

$$v_y = \frac{\gamma}{4\pi Q(1 + \alpha^2 \eta^2 / Q^2)} \int (\partial_x h)(1 - m_z) \, dx \, dy \qquad \text{and} \qquad v_x = -\frac{\eta \alpha}{Q} v_y \ , \tag{6}$$

which is in agreement with the results presented in Ref. [3] for the case $\alpha = 0$. For a skyrmion lattice, the dynamics is essentially of the same form.

In a circular field gradient $\mathbf{h} = h(r)\mathbf{e}_z$, the angular velocity $\omega$ can be defined as

$$\omega = \frac{\gamma}{4\pi Q(1+\alpha^2\eta^2/Q^2)}\frac{1}{r}\int(\partial_r h)(1-m_z)\,dx\,dy\ . \tag{7}$$

Numerical simulations based on an atomistic model with a 2D mesh were performed in order to study the skyrmion dynamics in a field gradient. In line with the micromagnetic energy [Eq. (2)], the corresponding Hamiltonian of the system is

$$\mathcal{H} = -J\sum_{\langle i,j\rangle}\mathbf{m}_i\cdot\mathbf{m}_j + \sum_{\langle i,j\rangle}\mathbf{D}_{ij}\cdot[\mathbf{m}_i\times\mathbf{m}_j] - \mu_s\sum_i(\mathbf{H}+\mathbf{h})\cdot\mathbf{m}_i, \tag{8}$$

where $\langle i,j\rangle$ represents a unique pair of lattice sites $i$ and $j$, $\mathbf{m}_i$ is the unit vector of the magnetic moment $\mu_s = -\hbar\gamma S_i$ with $\mathbf{S}_i$ being the atomic spin and $\gamma(>0)$ the gyromagnetic ratio. The DMI vector $\mathbf{D}_{ij}$ can be written as $\mathbf{D}_{ij} = \tilde{D}\hat{\mathbf{r}}_{ij}$, where $\tilde{D}$ is the DMI constant and $\hat{\mathbf{r}}_{ij}$ is the unit vector between $\mathbf{S}_i$ and $\mathbf{S}_j$.

In this work, a circular mesh with radius $R = 200$ is used. To reduce the simulation time, $\tilde{D}/J = 0.27$ is selected. The static field is chosen to be $H_z/J\mu_s = 0.04$ and the field gradient is a Gaussian function: $h/J\mu_s = 0.015e^{-4r^2/R^2}$. The simulation results for $\alpha = 0$ (no damping) of the skyrmion lattice rotation dynamics are shown in Supplementary Movie 4. The skyrmions are continuously rotating on circular trajectories. The angular velocity depends on the distance from the centre of the field gradient. On the other hand, by introducing damping, the skyrmions rotate on spiral trajectories, gradually moving towards the boundary of the sample, as shown in Supplementary Movie 5.

**Supplementary Methods 2. Sample and sample environment**

Single crystal $Cu_2OSeO_3$ is grown by chemical vapour transport method, as reported previously [4]. The (001)-oriented bulk crystal is characterised by single-crystal x-ray diffraction and electron backscatter diffraction, in order to to confirm the high quality of the single-crystalline and monochiral material [5]. The (001) scattering surface is subsequently polished using standard mechanical polishing, in order to be able to acquire a clean magnetic diffraction signal. The sample has a well-defined rectangular shape, measuring $4 \times 3$ mm$^2$, and it is placed in the centre of the field-gradient setup.

The field configuration is described by a vector field of the form $\mathbf{B}(\mathbf{r}) = [0, 0, h(\mathbf{r})]$. In other words, only the out-of-plane component of the magnetic field $h$ is present at the surface of the sample, while the amplitude of $h$ is concentrically decreasing away from the centre. In this configuration, an in-plane gradient of the form $\partial h/\partial r$ is obtained, with $r$ being the distance from the centre.

Experimentally, such a field configuration can be realised using a pair of cylindrical magnets of a size and relative distance well-matched to the sample size. Supplementary Figure 1a shows the in situ measured field distribution, and the position of the sample (marked by the white rectangle). As can be seen, only the magnetic field in the sample plane only has an out-of-plane component. Further, the magnetic field is strongest in the centre (35.5 mT), and it radially decays towards the boundary of the sample (34.1 mT). The field values are chosen such that the covered field range always stabilises the skyrmion lattice phase at 57 K for $Cu_2OSeO_3$ [6, 7]. Consequently, neither the helical nor the conical phase exist under these conditions. Supplementary Figure 1b shows the $h(r)$ profile across the centre. The field configuration can be fitted with a parabolic function, written as $h(r) = p_1 r^2 + p_2 r + p_3$, where $p_1$, $p_2$ and $p_3$ are fitting parameters.

Nevertheless, note that the sample only covers the very centre of the field gradient, as shown in Supplementary Figure 1a. Within this region, the $h(r)$ profile can be approximated by a linear function $h(r) = ar + b$, as indicated by the blue lines in Supplementary Figure 1b. This leads to a constant gradient $\partial h/\partial r = \text{const.}$, and thus a constant linear velocity [see Ref. [3], Eq. (28)]. Consequently, the angular velocity decreases with increasing distance from the centre as:

$$\omega \propto r^{-1} \quad . \tag{9}$$

**Supplementary Methods 3. Resonant elastic x-ray scattering setup**

Resonant elastic x-ray scattering (REXS) of $Cu_2OSeO_3$ in the reflection geometry is performed in the RASOR diffractometer at beamline I10 (Diamond Light Source, UK). The incident beam is horizontally polarised ($\sigma$-polarisation), and tuned to the Cu $L_3$ edge at 931.25 eV. The detailed REXS setup is described in detail elsewhere [5, 8]. In a radially-decaying field configuration, as shown in Supplementary Figure 1a, the skyrmion lattice phase is formed and rotating. This is manifested as multiple sets of six-fold-symmetric mag-

netic satellite peaks surrounding the (0,0,1) structural peak, which can be captured using a CCD camera.

The real-time measurement of the skyrmion lattice peak rotation is performed stroboscopically by switching a fast x-ray beam shutter in path of the incident light. For each exposure, the shutter is open for 2 ms. The scattered light is subsequently captured by the CCD camera. For our real-time REXS measurement, a standard 'rocking-scan-type' reciprocal space map is not feasible due to the desired time resolution. Therefore, for capturing the skyrmion rotation, the scattering angle of the detector is fixed to 96.5°, and the goniometer angle is optimised at one fixed angle for which one can observe all skyrmion lattice peaks surrounding the (0,0,1) peak. As a single goniometer angle cannot satisfy the perfect diffraction conditions for all skyrmion lattice satellite peaks, some of the observed magnetic peaks are weaker [5, 8]. More specifically, in the CCD camera image, the lower part of the magnetic signal is brighter than the upper part. In other words, during the rotation, the measured brightness of a specific skyrmion lattice peak varies depending on its position on the camera. Nevertheless, this effect does not compromise our quantitative REXS analysis, as we integrate the total magnetic diffraction cross-section over all six peaks belonging to one skyrmion lattice domain. In this case, the calculated total cross-section is a linear function of the measured signal. Note that the measured total magnetic diffraction intensity is weaker than expected from theoretical calculations, which assume perfect diffraction conditions for all six skyrmion lattice peaks in a single skyrmion lattice domain.

**Supplementary Methods 4. Resonant elastic x-ray scattering calculation**

The REXS scattering form factor in the electric-dipole approximation takes the form [8–11]:

$$f_n = \mathfrak{f}_0 \left(\epsilon_{\mathrm{s}}^* \cdot \epsilon_{\mathrm{i}}\right) - i\mathfrak{f}_1 \left(\epsilon_{\mathrm{s}}^* \times \epsilon_{\mathrm{i}}\right) \cdot \mathbf{m}_n \,, \tag{10}$$

where $\mathbf{m}_n$ is the magnetic moment that is carried by the magnetic ion at site $n$, $\epsilon_{\mathrm{i}}$ and $\epsilon_{\mathrm{s}}$ are the polarisation unit vectors of the incident and outgoing x-rays, and the asterisks denotes the complex conjugate. The $\mathfrak{f}_0$ and $\mathfrak{f}_1$ terms are the charge and the linear magnetic part of the energy-dependent resonance amplitude, respectively. In Eq. (10) we have neglected the term that is quadratic in the magnetisation as it is much smaller than the leading terms, and which gives rise to higher-order effects. The complex amplitudes $\mathfrak{f}_0$ and $\mathfrak{f}_1$ are energy-dependent. Here we take them as constant since in our numerical calculations the photon

energy is not varied.

The magnetic diffraction intensity at $\mathbf{Q}$ is then given by:

$$\begin{aligned} I(\mathbf{Q}) = & \frac{1}{2}|F_1|^2(P_0+P_1)|\mathbf{k}_\mathrm{s}\cdot\mathbf{M}(\mathbf{Q})|^2 \\ & + \frac{1}{2}|F_1|^2(P_0-P_1)\left[|\mathbf{k}_\mathrm{i}\cdot\mathbf{M}(\mathbf{Q})|^2+|(\mathbf{k}_\mathrm{s}\times\mathbf{k}_\mathrm{i})\cdot\mathbf{M}(\mathbf{Q})|^2\right] \\ & - |F_1|^2\mathrm{Re}\left[(P_2+iP_3)(\mathbf{k}_\mathrm{s}\cdot\mathbf{M}^*(\mathbf{Q}))(\mathbf{k}_\mathrm{s}\times\mathbf{k}_\mathrm{i})\cdot\mathbf{M}(\mathbf{Q})\right] \quad , \end{aligned} \tag{11}$$

where $F_1$ is the energy-dependent resonant term, and $\mathbf{M}(\mathbf{Q})$ is the Fourier transform of the real-space magnetic moment modulation $\mathbf{m}(\mathbf{r})$ at $\mathbf{Q}$. Note that $\mathbf{k}_\mathrm{i}$ and $\mathbf{k}_\mathrm{s}$ are tuned to fulfil the diffraction condition for $\mathbf{Q}$ at the scattering angle of $2\alpha$. $\mathbf{k}_\mathrm{i} = k(\cos\alpha, 0, \sin\alpha)$, $\mathbf{k}_\mathrm{s} = k(\cos\alpha, 0, -\sin\alpha)$, $\mathbf{k}_\mathrm{s}\times\mathbf{k}_\mathrm{i} = k(0, -2\cos\alpha\,\sin\alpha, 0)$, where the magnitude of the x-ray wavevector, $k$, relates to the photon energy [$k = 2\pi/\lambda = E/(\hbar c)$]. In the Poincaré-Stokes representation, the polarisation of the incident x-rays is characterised by $\mathbf{P} = (P_0, P_1, P_2, P_3)$. For horizontally polarised light, $P_0 = 1$, $P_1 = 1$, $P_2 = 0, P_3 = 0$. This immediately leads to:

$$I(\mathbf{Q})^\sigma = |F_1|^2|\mathbf{k}_\mathrm{s}\cdot\mathbf{M}(\mathbf{Q})|^2 \ . \tag{12}$$

In the case of rotating skyrmions, the single-domain skyrmion lattice breaks up into circular tracks with different radius, as a consequence of Eq. (7). Therefore, the individual skyrmion lattice domain has ring shape, which is the domain shape used in our REXS calculations, shown in Fig. 3b in the main text and in Supplementary Figure 2. Such ring track domains are described by their radius $r$ and width $dr$. It gives rise to six skyrmion lattice peak, located at $\mathbf{Q}_1, \mathbf{Q}_2, ..., \mathbf{Q}_6$. We measure the total magnetic diffraction intensity from all six peaks, i.e., $I^\sigma_\mathrm{SkX} = \sum_{i=1}^{6} I(\mathbf{Q}_i)$. The resulting relationship between $I^\sigma_\mathrm{SkX}$ and $r$, plotted in Fig. 3b in the main text, is given by:

$$I^\sigma_\mathrm{SkX} \propto r^2 \ . \tag{13}$$

In other words, the total diffraction intensity from each set of the six-fold-symmetric skyrmion lattice peaks directly relates to the domain radius. Together with Eq. (8), one expects the relationship between $\omega$ and $I^\sigma_\mathrm{SkX}$ to be:

$$\omega \propto {I^\sigma_\mathrm{SkX}}^{-1/2} \quad . \tag{14}$$

This is in excellent agreement with our measured data, as shown in Fig. 3c in the main text.

In order to further confirm this finding, we performed additional REXS measurements on the static (non-rotating), single domain skyrmion lattice state, stabilised with a uniform magnetic field. Using $\sigma$-polarised light, we can vary the beam size, which essentially changes the probing area. The results are shown in Supplementary Figure 3. The smallest beam possible size is 20 $\mu$m in diameter, using a pinhole. The reciprocal space map of the skyrmion lattice peak is shown in Supplementary Figure 3a, from which $I^{\sigma}_{\mathrm{SkX}}$ can be extracted. At the identical experimental conditions, we then subsequently increased the beam size to become a square beam with the following dimensions: $0.1 \times 0.1$ mm$^2$, $0.2 \times 0.2$ mm$^2$, $0.5 \times 0.5$ mm$^2$ and $1 \times 1$ mm$^2$. The experimental relationship between $I^{\sigma}_{\mathrm{SkX}}$ and the skyrmion lattice area is plotted in Supplementary Figure 3d (blue dots). The red curve shows a fit to the data with $I^{\sigma}_{\mathrm{SkX}} \propto S^2$. Therefore, we can confirm from both, experiment and theoretical calculation, that the $I^{\sigma}_{\mathrm{SkX}} \propto S^2$ relationship holds.

Next, let us take a closer look at the ring-shaped domains. Hereby, we assume that during the field-gradient-induced skyrmion lattice rotation, the entire sample (modelled as a disk) breaks into tracks, i.e., non-overlapping circular domain segments of finite width. Let us further assume that all tracks share the same width $dr$. Then the area of each track has an area of $S(r) = 2\pi r dr$. The diffraction intensity from that domain then scales with $S^2$, as shown above. Since $S$ is linear in $r$, and due to the fixed width $dr$, we obtain $I^{\sigma}_{\mathrm{SkX}} \propto r^2$.

Finally, we will show that the diffraction intensity also scales with $dr^2$. As shown in Supplementary Figures 4a and 4b, a disk-shaped skyrmion lattice domain again leads to the $I^{\sigma}_{\mathrm{SkX}} \propto S^2$ relation, which is consistent with the arguments above. Next, suppose such a long-range-ordered single domain breaks up into ring-shaped tracks, as shown in Supplementary Figure 4c. If the distance $r$ is fixed, clearly the domain size linearly scales with $dr$. This leads to the results shown in Supplementary Figure 4d, in which $I^{\sigma}_{\mathrm{SkX}} \propto dr^2$.

**Supplementary Methods 5. Exclusion of x-ray-induced effects**

There are several clear experimental pieces of evidence that directly prove that the impinging x-rays do not play a role for the induced skyrmion lattice rotation:

(1) A possible x-ray-induced effect could be the generation of a temperature gradient, therefore producing a magnon current that directly drives the single-domain skyrmion

lattice rotation. This mechanism would be similar to the one reported in Lorentz transmission electron microscopy imaging reported in Ref. [12]. However, in our experiment, the x-ray beam is not always impinging on the sample. Instead, there is a shutter installed that blocks the incoming beam and it is normally closed. For each snapshot, the shutter only opens for 2 ms during the exposure before it is closed again for at least 5 seconds. Therefore, the total heat that could be possibly deposited by the x-rays is minimised.

(2) We found very compelling evidence that the skyrmion lattice rotation is purely induced by the magnetic field gradient. First, we used highly uniform magnets. In such a setup, the single-domain, six-fold-symmetric REXS pattern is stabilised without any signs of rotation at all, regardless of the exposure time and exposure frequency, as shown in Fig. 2h in the main text. Once we switch on the field gradient, we immediately observe that the REXS pattern starts to rotate. Vice versa, once the field gradient is switched off, the REXS pattern returns to be static and stable.

(3) If the skyrmion rotation would be induced by the impinging x-rays, the system should relax to the static state after a reasonably long waiting time. Therefore, we kept the beam shutter closed for more than 5 hours, while the field gradient was applied. We then took one snapshot by opening the shutter for 2 ms. This image shows an washed-out pattern, suggesting that the skyrmion lattice is — and has been — rotating, even without the x-ray beam.

(4) If the rotation is related to the short-pulse impinging x-rays, the rotation mode should show a correlation with the exposure time and exposure frequency. However, by systematically varying the exposure time (ranging from 2 ms to 1 s) and exposure frequency, we found no correlation between these parameters and the rotation mode. We also did pump-probe-type tests using the same setup, i.e., using 2 ms exposure x-rays as a pump, and delay a certain time $t_d$ before opening the shutter to take a snapshot (probe). We varied $t_d$ from 5 to 60 s, and analysed how the probed snapshots are related in time. However, we found that the rotating skyrmions in the probed images appear to have random phases during the rotation. This proves that the rotation mode is not correlated with the x-rays at all.

(5) If the rotation is related to the x-ray-induced temperature gradient, by increasing the incoming x-ray flux, the temperature gradient should be increasing as well, leading to a different angular-velocity-intensity relationship. However, we found that the $\omega \propto {I^{\sigma}_{\mathrm{SkX}}}^{-1/2}$ relation is valid for varying x-ray flux, as well as for varying beam size. This excludes that the observed rotation is not due to the temperature gradient induced by the x-rays.

(6) As discussed in Ref. [12], the temperature-gradient-induced skyrmion lattice rotation should yield a universal angular velocity within one domain, where the gradient centre is at the domain centre. For our setup, only one gradient centre should exist, which coincides with the centre of the beam impinging on the sample. Consequently, one should expect that only one set of the six-fold-symmetric peaks collectively rotates. This clearly contradicts with our observations.

These observations suggest that the incoming x-rays have no significant effects on the skyrmion rotation dynamics in field gradients in our experimental setup.

**Supplementary Methods 6. Discussion of the potential device performance and scalability**

In the following, we will discuss the velocity of magnetic-field-induced skyrmion motion in the context of potential device applications, and the scalability of the field gradient scheme. Note that the following discussion is aimed at defining fundamental boundaries for the field gradient scheme. The detailed design, performance, and structural optimization goes far beyond the scope of this paper.

As shown in Supplementary Figure 5, the direct $\omega(r)$ relationship can be extracted based on the existing data and the well-established model. Supplementary Figure 5b shows the overview plot of the $\omega(r)$ relationship, which follows the inverse proportionality $\omega \propto r^{-1}$. A close-up for small $r$ is shown in Supplementary Figure 5a, and for large $r$ in Supplementary Figure 5c (note the different units of $r$). For a circular racetrack with the ring radius of ~500 nm, the angular velocity reaches almost $2\pi\,\mathrm{s}^{-1}$.

We can also evaluate the field-induced linear velocity in the linear racetrack design. For the outermost track, $r \approx 0.5$ mm, while $\omega \approx 0.5^\circ\,\mathrm{s}^{-1}$. This leads to the universal linear velocity $v = 2\pi\omega r \approx 1.6 \times 10^6$ $\mathrm{nm\,s^{-1}}$, which therefore makes it a promising method

compared to STT-driven skyrmions. Note that this linear velocity is the same for all domain tracks due to the $\omega \propto r^{-1}$ relationship. This is equal to the scenario of a linear racetrack with a linear field gradient (as shown in Fig. 1a (main text) and in Supplemental Movie 10). On the other hand, for typical spin-transfer-torque-driven magnetic domain wall motion devices, the domain walls obtain a speed of the order of $10^4$ nm s$^{-1}$ for a current density of 100 A cm$^{-2}$. Moreover, the typical skyrmion lattice moving velocity is also at this level. For example, a skyrmion lattice velocity of $v \approx 10^5$ nm s$^{-1}$ is observed in FeGe above the critical current density of 100 A cm$^{-2}$ [13].

Most importantly, the skyrmion velocity can be further improved in a straightforward way. It is clear from Eq. (2) in the main text that increasing the field gradient will linearly increase the moving velocity. For example, here we applied a very small field gradient of 0.1 mT mm$^{-1}$. It is fairly easy to fabricate an optimised field gradient source that enhances the gradient to about 10 mT mm$^{-1}$. In this case, the moving velocity will increase by two orders of magnitude. Therefore, field-gradient-induced skyrmion motion is a very promising way to manipulate nonvolatile data based on magnetic skyrmions, both for 1D and 2D racetrack memory, and independent of the type of skyrmion and host material.

For a fair comparison between the different racetrack design velocities, the current-normalised velocity (i.e., the velocity per applied current density) has been calculated. For a reasonable current density of $5 \times 10^7$ A cm$^{-2}$, the normalized velocity in case of domain-wall racetracks is ∼1 m s$^{-1}$ [14], that of spin-orbit-torque driven skyrmions in multilayers ∼100 m s$^{-1}$ [15, 16], that of spin-transfer-torque driven skyrmions in helimagnets ∼30 m s$^{-1}$ [13, 17], compared to ∼100 m s$^{-1}$ for our scheme (for 60 Oe $\mu$m$^{-1}$). In comparison with current-induced memory shift-register schemes, the field-gradient-induced scheme is in fact very promising for actual device applications.

Finally, we will comment on device scaling issues for field-gradient-induced skyrmion motion schemes. The size of skyrmions in helimagnets is generally smaller compared to skyrmions in multi-layered systems. The main practical issue for designing a device lies in the provision of the magnetic field gradient. A field gradient (the handle for controlling the motion) can be simply provided by a combination of miniaturized electromagnets. Note that this is fundamentally different from the Oersted-field-switching scheme in magnetic tunnel junction arrays. In magnetic tunnel junction arrays, each bit is operated by the

combined local Oersted fields of the write lines, thus compromising the storage density and energy efficiency. However, in the shift-register memory scheme, all bits share the same coil. Therefore, the field-gradient control coil can be orders of magnitude larger than a single bit and does not have to be scaled as the bit density goes up. If, for example, the common spiralling racetrack contains 1000 bits, we would need a 2-$\mu$m-diameter disk for $Cu_2OSeO_3$. Therefore, the field-gradient element can be larger than 2 $\mu$m in size, which is technologically feasible. Moreover, the larger the racetrack, the less the field-gradient coils have to be miniaturised. Consequently, this will not compromise the storage density.

**Supplementary Methods 7. Discussion of the energy source of skyrmion motion**
With regards to the energetics of the operation, i.e., the skyrmion motion in a magnetic field gradient, we have to stress that the applied magnetic field does not carry out any work on the skyrmions in the absence of damping. The skyrmions will simply follow contour lines of constant force. The foundation for this has been developed by Thiele and it has been discussed in great detail in, e.g., Ref. [18]. The treatment by Komineas and Papanicolaou excludes the presence of magnons due to the added complexity. Following their approach, we performed numerical simulations based on an atomistic model with a 2D mesh in order to study the skyrmion dynamics in a field gradient. The results are shown in the Supplementary Movies. In this scenario, the damping constant plays a central role for the long-term (> hours) dynamics of the system. Due to the very low spin wave damping observed in the $Cu_2OSeO_3$ system [19], the system will be able continuously rotate over a long period of time. Compared to domain wall motion it is also worth noting that only skyrmions offer flexibility of the trajectories and extremely small depinning currents [20], making the magnetic gradient drive possible.

Note, however, that the question what supplies the skyrmions with kinetic energy in the first place is meaningless in the framework of the Thiele equation in the slow-dynamics limit, as the skyrmion has no mass, and therefore, according to $E_{\rm kin} = \frac{1}{2}mv^2$, also no kinetic energy [21]. It has been discussed by some that there is a fluctuation-based mass possible for skyrmions, however, this is at this point an unproven hypothesis.

It is most plausible that it is the presence of magnons which initially start the skyrmion motion. It is important to note that the magnons do not have to be in the form of a (directed) current or flow resulting from a well-defined temperature gradient to drive skyrmion

motion. Generally, any arbitrarily moving magnon present at finite temperature can drive skyrmion motion. The existence of this process has been experimentally well-established by the rotation experiments of Tokura's group [12]. In their work they state that the rotation is driven solely by thermal fluctuations. It is nevertheless very important to bear in mind the differences. Whereas in Lorentz transmission electron microscopy it is the electron probe itself that causes a temperature gradient, and if this temperature gradient is large enough, a skyrmion lattice rotation occurs, whereas in our case it is solely the magnetic field that is the switch that controls the rotation. We experimentally showed that if this is removed, the rotation stops, i.e., thermal effects and skyrmion motion are uncorrelated. Our statement about the temperature homogeneity across our samples, i.e., that within the resolution of our temperature sensor ($\pm$10 mK) we cannot detect a temperature gradient, supports our claim that we do not have a directed magnon current that drives the rotation of the skyrmion lattice, however, it will likely assist it.

## Supplementary References


[1] A. A. Thiele, "Steady-state motion of magnetic domains," Phys. Rev. Lett. **30**, 230–233 (1973).

[2] K. Everschor, M. Garst, B. Binz, F. Jonietz, S. Mühlbauer, C. Pfleiderer, and A. Rosch, "Rotating skyrmion lattices by spin torques and field or temperature gradients," Phys. Rev. B **86**, 054432 (2012).

[3] S. Komineas and N. Papanicolaou, "Skyrmion dynamics in chiral ferromagnets," Phys. Rev. B **92**, 064412 (2015).

[4] M. Belesi, I. Rousochatzakis, H. C. Wu, H. Berger, I. V. Shvets, F. Mila, and J. P Ansermet, "Ferrimagnetism of the magnetoelectric compound $Cu_2OSeO_3$ probed by $S^{77}$-NMR," Phys. Rev. B **82**, 094422 (2010).

[5] S. L. Zhang, A. Bauer, D. M. Burn, P. Milde, E. Neuber, L. M. Eng, H. Berger, C. Pfleiderer, G. van der Laan, and T. Hesjedal, "Multidomain skyrmion lattice state in $Cu_2OSeO_3$," Nano Lett. **16**, 3285–3291 (2016).

[6] T. Adams, A. Chacon, M. Wagner, A. Bauer, G. Brandl, B. Pedersen, H. Berger, P. Lemmens, and C. Pfleiderer, "Long-wavelength helimagnetic order and skyrmion lattice phase in $Cu_2OSeO_3$," Phys. Rev. Lett. **108**, 237204 (2012).

[7] S. Seki, X. Z. Yu, S. Ishiwata, and Y. Tokura, "Observation of skyrmions in a multiferroic material," Science **336**, 198–201 (2012).

[8] S. L. Zhang, A. Bauer, H. Berger, C. Pfleiderer, G. van der Laan, and T. Hesjedal, "Resonant elastic x-ray scattering from the skyrmion lattice in $Cu_2OSeO_3$," Phys. Rev. B **93**, 214420 (2016).

[9] J. P. Hannon, G. T. Trammell, M. Blume, and D. Gibbs, "X-ray resonance exchange scattering," Phys. Rev. Lett. **61**, 1245–1248 (1988).

[10] M. Blume and D. Gibbs, "Polarization dependence of magnetic x-ray scattering," Phys. Rev. B **37**, 1779–1789 (1988).

[11] G. van der Laan, "Soft x-ray resonant magnetic scattering of magnetic nanostructures," C. R. Physique **9**, 570–584 (2008).

[12] M. Mochizuki, X. Z. Yu, S. Seki, N. Kanazawa, W. Koshibae, J. Zang, M. Mostovoy, Y. Tokura, and N. Nagaosa, "Thermally driven ratchet motion of a skyrmion microcrystal and topological magnon Hall effect," Nat. Mater. **13**, 241–246 (2014).

[13] X. Z. Yu, N. Kanazawa, W. Z. Zhang, T. Nagai, T. Hara, K. Kimoto, Y. Matsui, Y. Onose, and Y. Tokura, "Skyrmion flow near room temperature in an ultralow current density," Nat. Commun. **3**, 988 (2012).

[14] S.-H. Yang, K.-S. Ryu, and S. Parkin, "Domain-wall velocities of up to 750ms$^1$ driven by exchange-coupling torque in synthetic antiferromagnets," Nat. Nanotechnol. **10**, 221–226 (2015).

[15] S. Woo, K. Litzius, B. Krüger, M.-Y. Im, L. Caretta, K. Richter, M. Mann, A. Krone, R. M. Reeve, M. Weigand, P. Agrawal, I. Lemesh, M.-A. Mawass, P. Fischer, M. Kläui, and G. S. D. Beach, "Observation of room-temperature magnetic skyrmions and their current-driven dynamics in ultrathin metallic ferromagnets," Nat. Mater. **15**, 501–506 (2016).

[16] K. Litzius, I. Lemesh, B. Krüger, P. Bassirian, L. Caretta, K. Richter, F. Büttner, K. Sato, O. A. Tretiakov, J. Förster, R. M. Reeve, M. Weigand, I. Bykova, H. Stoll, G. Schütz, G. S. D. Beach, and M. Kläui, "Skyrmion Hall effect revealed by direct time-resolved X-ray microscopy," Nat. Phys. **13**, 170–175 (2017).

[17] J. Iwasaki, M. Mochizuki, and N. Nagaosa, "Universal current-velocity relation of skyrmion motion in chiral magnets," Nat. Commun. **4**, 1463 (2013).

[18] S. Komineas and N. Papanicolaou, "Skyrmion dynamics in chiral ferromagnets under spin-transfer torque," Phys. Rev. B **92**, 174405 (2015).

[19] I. Stasinopoulos, S. Weichselbaumer, A. Bauer, J. Waizner, H. Berger, M. Garst, C. Pfleiderer, and D. Grundler, "Linearly polarized GHz magnetization dynamics of spin helix modes in the ferrimagnetic insulator $Cu_2OSeO_3$," Sci. Rep. **7**, 7037 (2017).

[20] A. Fert, N. Reyren, and V. Cros, "Magnetic skyrmions: advances in physics and potential applications," Nat. Rev. Mater. **2**, 17031 (2017).

[21] M. Garst, "Topological skyrmion dynamics in chiral magnets," in *Topological Structures in Ferroic Materials: Domain Walls, Vortices and Skyrmions* (Springer International Publishing, Cham, 2016) pp. 29–53.
