# Supplementary material for: Manipulation of skyrmion motion by magnetic field gradients
Source: Nat Commun. 2018 May 29;9:2115. doi: 10.1038/s41467-018-04563-4 (PMC5974091; doi:10.1038/s41467-018-04563-4)
Supplement: Supplementary file 2 — Descriptions of Additional Supplementary Files [file 41467_2018_4563_MOESM2_ESM.pdf]

Descriptions of Additional Supplementary Files

File Name: Supplementary Movie 1
Description: Experimental REXS time-lapse video of skyrmion lattice rotation at the onset of rotation. Real-time snapshots of the REXS pattern showing the rotation of the skyrmion lattice domains in a field gradient. The experimental geometry is shown in Fig. 2f in the main text. The temperature is kept at 57 K and the incident beam is horizontally polarised with a photon energy of 931.25 eV. Each 'snapshot' is a CCD camera image exposed for 2 ms, taken every 5 s. The movie shows the start of the skyrmion rotation, i.e., after the sample was field-cooled down from 65 K to 57 K, and continues for ∼16 min.

File Name: Supplementary Movie 2
Description: Experimental time-lapse video of skyrmion lattice rotation obtained by REXS 25 min after the start of the rotation. This movie is a continuation of the one shown in Supplementary Movie 1, starting ∼25 min after the beginning of skyrmion rotation in a field gradient.

File Name: Supplementary Movie 3
Description: Simulation of the REXS pattern of the skyrmion lattice rotation. On the left-hand side, an animation of the rotating skyrmion lattice is shown, in which the skyrmion lattice fragments into discrete tracks. Note that such real-space configuration is not from micromagnetic simulation results. On the right-hand side, the corresponding REXS pattern, using σ-polarised resonant x-rays at Cu L3 edge is shown, in which six-fold sets of dimmer spots rotate faster than sets of brighter spots. The REXS calculation details are described in Supplementary Methods 4.

File Name: Supplementary Movie 4
Description: Micromagnetic simulation of the skyrmion lattice dynamics in a linear field gradient. Subjecting a skyrmion lattice to a linear magnetic field gradient (increasing from left to right) forces the skyrmions to move along the (vertical) lines of constant field upwards. The micromagnetic simulation parameters can be found in

File Name: Supplementary Movie 5
Description: Micromagnetic simulation of the skyrmion lattice dynamics in a radial field gradient (undamped case; $\alpha = 0$). The simulation shows the counterclockwise rotation of the skyrmion lattice in a field gradient that decreases from the centre to the boundary of the disk. As before, the skyrmions move along the lines of constant field (which are now circular).

File Name: Supplementary Movie 6
Description: Micromagnetic simulation of the skyrmion lattice dynamics in a radial field gradient (undamped case; $\alpha = 0.1$). By including damping, the rotating skyrmions follow spiral trajectories, pushing them towards the perimeter of the disk.

File Name: Supplementary Movie 7
Description: Micromagnetic simulation of the dynamics of a single skyrmion as a function of field parameters in a radial field gradient. The simulation shows the results for a negative field with a positive gradient in the top left, a negative field and a negative gradient in the top right, a positive field and a positive gradient in the bottom left, and a positive field and a negative gradient in the bottom right corner.

File Name: Supplementary Movie 8

Description: Micromagnetic simulation of the dynamics of two skyrmions in a radially linear field gradient. As can be seen, the angular velocity of the outermost skyrmion is slower than that of the inner one. In this case, the relationship $\omega \sim r^{-1}$ holds.

File Name: Supplementary Movie 9
Description: Micromagnetic simulation of the skyrmion dynamics in a ring. The movie shows the rotation of a chain of skyrmions in a one-skyrmion-wide ring in a radial field gradient.

File Name: Supplementary Movie 10
Description: Micromagnetic simulation of the skyrmion dynamics in a stripe. The movie shows the movement of skyrmions in a one-skyrmion-wide strip in a linear field gradient.
